# Sex Differences in Scalp‐to‐Cortex Distance: Implications for Transcranial Magnetic Stimulation Efficacy in Alcohol Use Disorder

**DOI:** 10.1111/acer.70358

**Published:** 2026-06-30

**Authors:** Kaitlin R. Kinney, Nathanial E. Stewart, Michiyah Kimber, Hannah E. DeMaioNewton, Jazmyne S. James, Nathan R. Luzum, Edward H. Ip, Hilary R. Smith, Drew D. Kiraly, Colleen A. Hanlon, Merideth A. Addicott

**Affiliations:** ^1^ Department of Translational Neuroscience Wake Forest University School of Medicine Winston‐Salem North Carolina USA; ^2^ Department of Psychiatry Wake Forest University School of Medicine, Atrium Wake Forest Baptist Health Winston‐Salem North Carolina USA; ^3^ Department of Biostatistics and Data Science Wake Forest University School of Medicine Winston‐Salem North Carolina USA; ^4^ Wake Forest Center for Addiction Research Wake Forest University School of Medicine Winston‐Salem North Carolina USA; ^5^ Department of Cancer Biology Wake Forest School of Medicine Winston‐Salem North Carolina USA; ^6^ BrainsWay Ltd Jerusalem Israel

**Keywords:** alcohol use disorder, electric field modeling, scalp‐to‐cortex distance, sex differences, transcranial magnetic stimulation

## Abstract

**Introduction:**

Transcranial magnetic stimulation (TMS) is a promising intervention for alcohol use disorder (AUD), yet its efficacy varies widely. Scalp‐to‐cortex (STC) distance influences the strength of the induced electric field, and preliminary evidence suggests sex‐specific craniofacial anatomy may contribute to STC variability. However, the implications of anatomical sex differences for TMS dosing in AUD remain unclear. This study examined whether STC distance and modeled electric field magnitude differ by sex, age, and alcohol use severity across common TMS targets.

**Methods:**

High‐resolution structural magnetic resonance imaging was acquired from 107 individuals with AUD (59 females, 48 males). Individualized finite‐element head models were generated using SimNIBS to calculate STC distance and electric field strength at four cortical targets: medial prefrontal cortex (Afz), dorsolateral prefrontal cortex (F3), ventromedial prefrontal cortex (Fp1), and motor cortex (C3). Linear mixed‐effects models assessed the effects of sex, age, and Alcohol Use Disorders Identification Test (AUDIT) score.

**Results:**

STC distance differed significantly across sites and was larger in males than in females, with sex differences strongest at Fp1 and C3. No sex differences were observed at Afz or F3. Higher AUDIT scores predicted greater STC distance independent of age. Electric field magnitude varied significantly across sites, with reduced values at midline regions (Afz, Fp1) relative to lateral sites (F3, C3). Electric field magnitude did not differ by sex overall. Age was associated with lower estimated electric field magnitude at the cortical surface. Exploratory sex‐stratified analyses indicated a steeper age‐related decline in females, particularly at medial prefrontal targets.

**Conclusions:**

Sex‐specific and age‐related anatomical differences influence cortical depth and effective stimulation strength in individuals with AUD. These findings highlight the need for individualized and potentially sex‐tailored TMS dosing strategies, particularly for midline prefrontal targets and for older females who may be more vulnerable to reduced TMS engagement.

## Graphical abstract

In individuals with alcohol use disorder, scalp‐to‐cortex distance varied by sex, alcohol use severity, and TMS target, while modeled electric field magnitude varied by target and age. These findings support individualized, anatomy‐informed TMS dosing strategies.

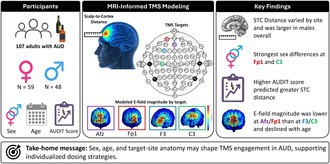

## 1 Introduction

Alcohol use disorders (AUDs) are highly prevalent, debilitating, and notoriously difficult to treat. Despite the availability of behavioral and pharmacological interventions, relapse rates remain high, with approximately 60% of individuals relapsing within 6 months of completing treatment (Bergmann et al. 2021; Kirshenbaum et al. 2009; Maisto et al. 2006). This underscores an urgent need for more effective therapeutic approaches for treating AUD. Notably, sex differences in AUD prevalence and outcomes have become increasingly evident. Over the past decade, the rate of AUD in females has surged by 83.7%, compared to a 34.7% increase in males (Grant et al. 2017). This trend is particularly concerning given that females tend to experience more severe health consequences from alcohol use at lower consumption levels (Baraona et al. 2001; Cherpitel and Ye 2014). Characterizing sex‐related factors that may influence treatment engagement and efficacy is, therefore, essential for advancing more effective interventions for both males and females with AUD.

While relapse patterns and sex differences in AUD have been most extensively examined in the context of behavioral and pharmacological treatments, neuromodulation interventions such as transcranial magnetic stimulation (TMS) are constrained by distinct biological mechanisms. TMS is a non‐invasive neuromodulation technique that uses electromagnetic induction to directly engage and depolarize neurons in targeted cortical circuits, making its efficacy particularly sensitive to individual differences in neuroanatomy and the biophysical properties of the head. It is FDA‐approved for the treatment of depression (O'Reardon et al. 2007), obsessive‐compulsive disorder (OCD) (Carmi et al. 2018, 2019), and smoking cessation (Zangen et al. 2021), and has shown potential in addressing a range of neuropsychiatric conditions including substance use disorders, post‐traumatic stress disorder, and anxiety‐related disorders (Hanlon et al. 2018; Kearney‐Ramos et al. 2018). Emerging research has been investigating how TMS might be leveraged to support individuals with AUD. Although more than 20 studies have examined TMS as a method to reduce alcohol consumption (Hanlon et al. 2018), many report modest effect sizes and substantial individual variability. This raises a critical question: *what factors could affect TMS's efficacy in AUD populations?*

One key factor may be an individual's scalp‐to‐cortex (STC) distance, which refers to the physical space between the TMS coil placement on the scalp and the underlying cortical target. The effectiveness of TMS depends on the strength of the electromagnetic field reaching the cortex, which diminishes rapidly with increasing distance (Maxwell 1861; Terao and Ugawa 2002). Although the magnetic field can penetrate the skull and induce an electrical current in cortical tissue, its intensity is attenuated by extracerebral structures such as the scalp, skull, and meninges (Klomjai et al. 2015). Consequently, individuals with greater STC distances may receive a weaker stimulation dose, potentially leading to reduced neuromodulatory effects (Hanlon et al. 2019; Kearney‐Ramos et al. 2018). Understanding and accounting for these anatomical differences is essential for optimizing TMS protocols and improving treatment outcomes.

A growing body of work in healthy and aging populations has demonstrated that STC distance varies systematically as a function of sex, age, and cortical region, with important implications for the effective dose of TMS (Lu et al. 2019, 2021; McCalley et al. 2026; Van Hoornweder et al. 2024). These studies have shown that anatomical variability differs substantially across frontal, motor, and midline targets, and that sex‐ and age‐related effects are not uniform across the cortex. Together, this literature has established STC distance as a critical determinant of TMS‐induced electric field strength in non‐clinical samples. However, the extent to which these anatomical patterns generalize to clinical populations characterized by alcohol‐related neurodegeneration remains unclear.

Chronic alcohol use is associated with cortical atrophy, particularly in prefrontal regions involved in executive function and impulse control (Cardenas et al. 2007; Durazzo et al. 2008; Zahr et al. 2017). These structural changes can increase the STC distance by altering the geometry of the brain–skull interface, thereby weakening the strength of TMS‐induced electric fields (Hanlon et al. 2019; McCalley and Hanlon 2021). Importantly, individuals with more severe AUD tend to exhibit greater cortical thinning (Demirakca et al. 2011; Durazzo et al. 2011; Segobin et al. 2014), which may further exacerbate STC distance‐related attenuation of stimulation efficacy. Because repetitive transcranial magnetic stimulation (rTMS), including intermittent theta burst stimulation (iTBS), relies on both precise targeting and sufficient field strength to modulate cortical activity, characterizing alcohol‐related changes in STC distance is critical for optimizing stimulation protocols. Whether sex‐ and age‐related differences in STC distance observed in healthy populations are preserved, amplified, or altered in AUD—and how such variability may interact with alcohol use severity to influence effective stimulation dose—has not been systematically examined.

Preliminary data from our lab suggest that sex‐specific craniofacial anatomy may play a role in STC variability. Females exhibited significantly shorter STC distances than males at the left medial prefrontal cortex (MPFC), but not at the left dorsolateral prefrontal cortex (DLPFC) or motor cortex (McCalley and Hanlon 2021). These differences are attributable to sex‐specific craniofacial anatomy. While females typically have sharper, more angular facial features, males exhibit a facial bone structure that is less angular and appears more rounded with a prominent brow ridge (Toledo Avelar et al. 2017). These skeletal distinctions, rather than differences in brain morphology, result in greater STC distances at the left MPFC in males, leading to weaker TMS‐induced electric fields in that cortical region (Hanlon and McCalley 2022). However, the initial study did not balance participants by sex, with females comprising only 17% of the AUD sample. This imbalance limits our understanding of how sex may influence STC distance and, by extension, TMS efficacy in individuals with AUD. Further investigation is needed to clarify how sex differences in anatomical variability may influence the delivery and effectiveness of TMS.

By systematically characterizing sex differences in STC distance across key TMS target sites, this study aims to inform the development of anatomically tailored stimulation protocols for individuals with AUD. Prior research suggests that sex‐specific craniofacial anatomy contributes to STC variability, particularly at midline prefrontal regions, though findings have been limited by small female sample sizes. Building on this work, we hypothesized that males would exhibit greater STC distances at the MPFC, specifically at both Fp1 and Afz, while no significant sex differences would be observed at the DLPFC (F3) or motor cortex (C3). While STC distance provides a structural proxy for TMS dosing, it does not directly quantify the strength of the electric field reaching cortical targets. To address this limitation, a secondary analysis was conducted using SimNIBS‐modeled electric field magnitude to evaluate how anatomical and demographic factors, including age, sex, and alcohol use severity, may influence the effective dose delivered to the cortex. This complementary analysis enhances the interpretability of STC findings and may help refine dosing strategies, particularly for female individuals with AUD, who remain underrepresented in research despite experiencing rising prevalence and heightened vulnerability to alcohol‐related harm.

## 2 Methods

This study is an observational secondary analysis of baseline neuroimaging data collected within a randomized, double‐blind, sham‐controlled clinical trial, for which the full protocol is published in *Trials* (Kinney et al. 2025).

### 2.1 Participants

One hundred seven individuals with AUD (59F, 48M) were recruited from the Winston‐Salem, North Carolina, community using digital and print materials, as well as internet postings of clinical studies conducted at Atrium Health Wake Forest Baptist Medical Center. Of these, 79 met criteria for severe AUD, 23 for moderate AUD, and five for mild AUD, based on DSM‐5 criteria. Inclusion criteria included (1) meeting DSM‐5 criteria for current AUD, (2) having an Alcohol Use Disorders Identification Test (AUDIT) score ≥ 8, and (3) having a desire to reduce or quit alcohol consumption. Exclusion criteria included: (1) current use of any psychoactive substance (excluding nicotine and cannabis) within the past 30 days, based on self‐report and verified via urine drug screen; (2) a DSM‐5 Axis I diagnosis of schizoaffective disorder or schizophrenia; (3) current suicidal ideation; (4) current use or initiation of medications known to affect alcohol intake or craving (e.g., disulfiram, naltrexone, acamprosate); (5) current enrollment in another form of treatment for AUD; (6) chronic migraines or elevated risk of seizure; (7) history of traumatic brain injury, stroke, or epilepsy; and (8) pregnancy, breastfeeding, ferromagnetic metal in the body, or severe claustrophobia. Each participant provided written informed consent prior to participation (*n* = 107). Experimental protocols were reviewed and approved by the Atrium Health Wake Forest Baptist Institutional Review Board and were performed in accordance with the Declaration of Helsinki on Ethical Principles for Medical Research. These participants represent a subset of individuals from a single‐site, double‐blinded, randomized, sham‐controlled clinical trial investigating the efficacy of iTBS targeting either the MPFC (Afz) or the left DLPFC (F3) for AUD. This subset completed baseline MRI scanning as part of a larger neuroimaging protocol designed to assess changes in alcohol consumption, craving, and brain reactivity to alcohol cues following 30 sessions of iTBS (Kinney et al. 2025).

### 2.2 Assessments

The present study utilized a subset of data from a larger clinical trial. The full assessment battery for the parent trial is described in detail in (Kinney et al. 2025). For the present analysis, we used baseline measures from the following assessments:

The Alcohol Use Disorders Identification Test (AUDIT) (Saunders et al. 1993) was administered at the initial screening visit to screen for risky or hazardous alcohol consumption. The AUDIT is a 10‐item questionnaire that assesses alcohol consumption patterns, drinking behaviors, and alcohol‐related problems over the past 12 months, with scores ranging from 0 to 40. DSM‐5 AUD diagnoses and severity classifications (mild, moderate, severe) were determined using a structured clinical interview assessing symptoms within the past 12 months. The Timeline Follow‐Back (TLFB) (Sobell and Sobell 1992) was administered at screening to evaluate drinking patterns over the previous 30 days. The TLFB uses a calendar‐based method to assess daily alcohol consumption, from which we derived the number of heavy drinking days (defined as ≥ 4 drinks for women, ≥ 5 drinks for men per day). The Alcohol Urge Questionnaire (AUQ) (Bohn et al. 1995) was administered at screening to measure alcohol craving. This 8‐item scale assesses the intensity of urges to drink, with higher scores indicating greater craving intensity. The Obsessive Compulsive Drinking Scale (OCDS) (Anton et al. 1995) was administered at screening to evaluate obsessive thoughts and compulsive behaviors related to alcohol use. The OCDS consists of 14 items yielding a total score and two subscales (obsessive and compulsive), with higher scores reflecting greater severity of alcohol‐related obsessions and compulsions. The Beck Depression Inventory (BDI) (Beck et al. 1996) was administered at the baseline MRI visit to assess depressive symptomatology. This 21‐item self‐report questionnaire measures the severity of depressive symptoms over the past 2 weeks, with total scores ranging from 0 to 63.

### 2.3 MRI Acquisition

All participants (*n* = 107) underwent high‐resolution (0.5 × 0.5 × 1.0 mm^3^) T1‐weighted structural MRI scanning on a Siemens Magnetom Skyra 3T scanner. Images were acquired using a magnetization‐prepared rapid gradient echo (MPRAGE) sequence (TR = 1480 ms; TE = 3.86 ms; flip angle = 12°; FoV = 24.5 cm; 192 slices).

### 2.4 Construction of Individual Head Models

Individual head‐mesh models were constructed using the “headreco” command within SIMNIBS version 3.2.5, which offers high accuracy in tissue segmentation and surface reconstruction (Nielsen et al. 2018). T1‐weighted anatomical images were segmented into gray matter, white matter, cerebrospinal fluid, skull, skin, and background using SPM12 (Penny et al. 2007). Surface mesh reconstructions (excluding background) were created with the Computational Anatomy Toolbox (CAT12; Gaser et al. 2024), and tetrahedral meshes were generated using Gmsh (Geuzaine and Remacle 2009).

### 2.5 Modeling TMS‐Induced Electric Fields and Scalp‐to‐Cortex Distance

**FIGURE 1:**
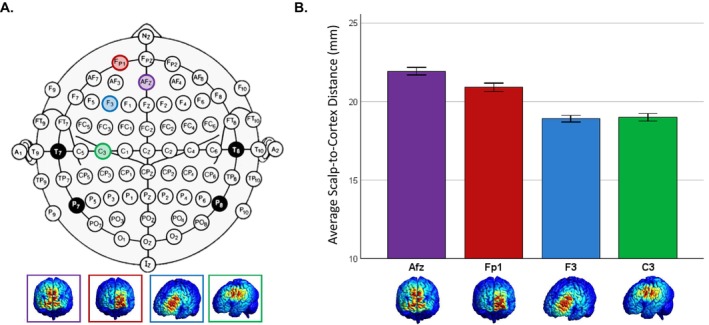
EEG electrode positions and scalp‐to‐cortex distances for each TMS targeting site. (A) Electric field modeling was performed at four EEG 10–20 system electrode positions commonly used as TMS targets: Afz (medial prefrontal cortex, MPFC; purple), F3 (left dorsolateral prefrontal cortex, DLPFC; blue), Fp1 (left ventromedial prefrontal cortex, vmPFC; red), and C3 (left motor cortex; green). Electrode positions are shown on a schematic EEG cap, with corresponding brain renderings below each site. (B) Average scalp‐to‐cortex distances (in mm) are shown for each stimulation site. There was a significant main effect of TMS site on scalp‐to‐cortex distance, *F*_(3,314.54)_ = 92.27, *p* < 0.001. Post hoc comparisons revealed significant differences between all sites (*p* < 0.001) except F3 and C3 (*p* > 0.999). Mean distances were: Afz = 21.92 mm (SE = 0.23), Fp1 = 21.03 mm (SE = 0.23), C3 = 19.03 mm (SE = 0.23), and F3 = 18.91 mm (SE = 0.23). Error bars represent ±1 standard error. See Figure S2A for boxplots illustrating the full data distribution.

TMS‐induced electric fields were modeled using SIMNIBS version 3.2.5 via the finite‐element method using the individualized head‐mesh models described above (Thielscher et al. 2015). Electric fields were modeled at four EEG 10–20 system electrode positions corresponding to common TMS targets: Afz (MPFC), F3 (left DLPFC), Fp1 (left vmPFC), and C3 (left motor cortex) (Figure 1A). Motor cortex stimulation was modeled at C3, consistent with standard TMS protocols that use the primary motor cortex to determine resting motor threshold (rMT) for dosing and calibration purposes (Rossini et al. 2015). This approach was applied uniformly across participants to maintain methodological consistency. Handedness was not systematically assessed in this sample. Although the motor cortex is not a therapeutic target for TMS in AUD, it is routinely stimulated to determine rMT, a standard metric used to guide TMS dosing. At Afz and Fp1, the coil was positioned parallel to the eyebrow, with the handle pointing upward. This orientation aligned the long axis of the figure‐of‐eight coil with the underlying gyrus. At F3 and C3, the coil was oriented at approximately 45° to the sagittal plane, with the handle pointing posteriorly, to induce a posterior–anterior electric field perpendicular to the central sulcus, which is optimal for motor cortex stimulation. All models were constructed using the MagVenture B70 butterfly‐cooled sham coil. For each participant, the rate of change of coil current (dI/dT) was individually calculated based on their motor threshold measured using the MagVenture X100 stimulator. The value was determined by linearly scaling the coil's maximum dI/dT (146 A/μs at 100% maximum stimulator output [MSO]) according to the percentage of MSO needed to trigger a motor response (Drakaki et al. 2022). In other words, the coil's maximum current change rate was adjusted for each person depending on how much stimulation was required to elicit a motor response in the right hand. This individualized approach ensured that the simulated electric fields reflected realistic and physiologically relevant stimulation intensities. Because the electric field magnitude was modeled using participant‐specific rMT‐scaled stimulation intensity, rMT was inherently incorporated into the outcome measure and was not included as a separate covariate in statistical analyses. Standard tissue conductivity values were applied: white matter = 0.125 S/m, gray matter = 0.275 S/m, CSF = 1.654 S/m, bone = 0.010 S/m, and scalp = 0.465 S/m (Opitz et al. 2011). Electric field maps were interpolated into both subject‐space and MNI‐space NIfTI formats. Figure S1 presents representative electric field models for males and females at each cortical target site. STC distance was extracted directly from the SimNIBS output, which computes the shortest distance from the center of the TMS coil to the cortical surface beneath each target. Specifically, STC distance was operationalized as the minimum Euclidean distance between the center of the TMS coil and the reconstructed cortical surface derived from individual head meshes. For each stimulation site, SimNIBS identifies the cortical surface vertex nearest to the coil center in three‐dimensional space, independent of local gyral folding or sulcal geometry. This approach yields a shortest‐path estimate that inherently accounts for cortical curvature, rather than relying on a fixed normal vector from the scalp or gray matter surface. At midline targets such as Afz, this procedure identifies the nearest cortical surface point across the medial wall, providing a consistent and anatomically grounded estimate of depth despite regional convolution. Because SimNIBS reports the coil‐to‐cortex distance, which includes the physical thickness of the coil housing, a fixed 4 mm offset, representing the approximate coil center‐to‐scalp distance, was subtracted from each measurement to obtain the true STC value at the cortical surface. The electric field magnitude at the top 1% of all values was also extracted directly from SimNIBS. This value, referred to as the 99th percentile or 99% peak electric field, represents the threshold below which 99% of simulated field magnitudes fall. It provides a robust estimate of peak stimulation intensity while minimizing the influence of numerical artifacts or mesh‐related outliers. Throughout the manuscript, ‘electric field magnitude’ refers to the modeled magnitude (V/m) of the TMS‐induced electric field estimated using SimNIBS and does not refer to stimulator output or motor‐threshold‐based dosing parameters.

### 2.6 Statistical Analysis

#### 2.6.1 Demographics and Behavior

Independent samples *t*‐tests were conducted in SPSS (v29) to assess sex differences across key variables, including age, BDI score, rMT, and alcohol‐related measures (AUDIT, OCDS total score, AUQ, and number of heavy drinking days). Prior to each *t*‐test, Levene's Test for Equality of Variances was assessed to determine whether the assumption of equal variances was met. Based on the results of Levene's Test, the appropriate *t*‐test results (equal or unequal variances assumed) were reported. All tests were two‐tailed, and statistical significance was set at *p* < 0.05.

#### 2.6.2 Primary Analysis: Scalp‐to‐Cortex Distance

A linear mixed‐effects model was conducted in SPSS (v29) to examine scalp‐to‐cortex distance across four anatomical sites (within‐subjects factor: Afz, F3, Fp1, C3) and sex (between‐subjects factor: male, female), controlling for age and AUDIT score. The mixed‐effects approach was used to account for the non‐independence of repeated measures within participants. The model included site and sex as fixed factors, age and AUDIT as covariates, and a site‐by‐sex interaction term. A random effect was included to account for within‐subject correlations. Restricted maximum likelihood (REML) estimation was used, with the Satterthwaite method for degrees of freedom approximation. Estimated marginal means and pairwise comparisons were conducted for post hoc tests using a Bonferroni‐adjusted correction set at *α* = 0.05.

##### 2.6.2.1 Model Diagnostics and Assumptions

Model assumptions were assessed through examination of residual plots and normality of random effects. The intraclass correlation coefficient (ICC) was calculated to quantify the proportion of variance attributable to between‐participant differences. Model fit was evaluated using pseudo‐*R* ^2^ measures, with marginal *R* ^2^ representing variance explained by fixed effects and conditional *R* ^2^ representing total variance explained by the full model.

#### 2.6.3 Secondary Analysis: Electric Field Modeling

A linear mixed‐effects model was conducted in SPSS (v29) to examine electric field magnitude (99th percentile value) across four anatomical sites (Afz, F3, Fp1, C3) and sex, controlling for age and AUDIT score. The model structure and estimation procedures were identical to those described in the primary analysis, including the use of REML estimation, Satterthwaite approximation for degrees of freedom, and Bonferroni‐adjusted post hoc comparisons. Model diagnostics also followed the same procedures outlined in the primary analysis.

In these primary and secondary models, age was included as a covariate to estimate overall effects of sex and stimulation site while accounting for age‐related variability. Sex‐by‐age interaction terms were not specified a priori due to limited power for higher‐order effects and were examined only in exploratory analyses when a significant main effect of age was observed. Accordingly, exploratory sex‐stratified models were conducted to further characterize age effects within each sex; sex was not included as a predictor in these stratified analyses because it has no variance within sex‐specific models.

#### 2.6.4 Exploratory Analysis

To further explore potential moderators of electric field magnitude, additional linear mixed‐effects models were conducted. Following initial inspection of a sex‐by‐age interaction, post hoc models were run separately for male and female participants. Each model included anatomical site (Afz, F3, Fp1, C3) as a within‐subjects factor and age and AUDIT score as covariates. Sex was excluded from these stratified models. Model structure and estimation procedures were consistent with those used in the primary and secondary analyses. To examine whether the AUDIT score moderated the relationship between age and electric field magnitude, an age‐by‐AUDIT interaction term was added to each sex‐specific model. Model diagnostics followed the same procedures outlined previously.

## 3 Results

### 3.1 Demographics and Behavior

Demographic and behavioral comparisons between male and female participants revealed no statistically significant differences across all measures. Specifically, there were no significant differences between males and females in age (*p* = 0.963), BDI score (*p* = 0.213), or rMT (*p* = 0.983). Measures of alcohol use and severity also showed no significant group differences, including AUDIT score (*p* = 0.608), OCDS total score (*p* = 0.166), AUQ score (*p* = 0.766), or number of heavy drinking days (*p* = 0.723). Demographics can be found in Table 1.

**TABLE 1:** Demographics.

	Females (*N* = 59)	Males (*N* = 48)	*p*‐value*
Age	44.36 ± 13.51	44.48 ± 13.57	0.963
Depression symptoms (BDI)	11.85 ± 9.01	9.63 ± 9.25	0.213
Race			
African American	8	8	0.629**
White	46	38	
Other	5	2	
Ethnicity			
Hispanic	5	6	0.495**
Non‐Hispanic	54	42	
Average resting motor threshold	50.62 ± 10.21	50.66 ± 8.61	0.983
Alcohol use			
Alcohol Use Disorders Identification Test Score	17.83 ± 5.34	18.38 ± 5.56	0.608
Obsessive Compulsive Drinking Scale	14.93 ± 4.77	13.65 ± 4.72	0.166
Alcohol Urge Questionnaire (AU Score)	21.08 ± 11.15	21.71 ± 10.26	0.766
Heavy drinking days last month	14.24 ± 9.99	15.05 ± 12.25	0.723

*Note:* No significant differences were observed between females and males across any demographic variables, based on independent samples *t*‐tests and chi‐squared tests, as appropriate. Values are presented as mean ± SD.

Abbreviations: AUDIT, Alcohol Use Disorders Identification Test; AUQ, Alcohol Urge Questionnaire; BDI, Beck Depression Inventory; OCDS, Obsessive Compulsive Drinking Score; rMT, resting motor threshold; SD, standard deviation.

* independent samples *t‐*test *p*‐value.

** Chi‐square *p*‐value.

### 3.2 Primary Analysis: Scalp‐To‐Cortex Distance

There was a significant main effect of TMS site on scalp‐to‐cortex distance, *F*_(3,314.54)_ = 92.27, *p* < 0.001 (Figure 1B). Post hoc pairwise comparisons revealed significant differences between all sites (*p* < 0.001) except F3 and C3 (*p* > 0.999). Mean distances were Afz = 21.92 mm (SE = 0.23), Fp1 = 21.03 mm (SE = 0.23), C3 = 19.03 mm (SE = 0.23), and F3 = 18.91 mm (SE = 0.23). For transparency, boxplots illustrating the full data distribution are shown in Figure S2A.

**FIGURE 2:**
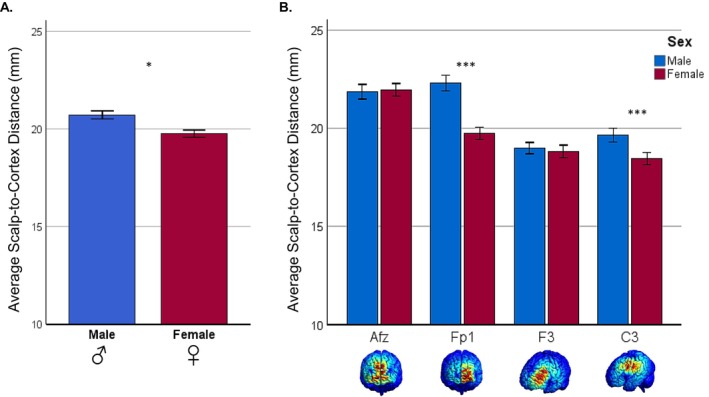
Sex differences in scalp‐to‐cortex distance across measurement sites. (A) Average scalp‐to‐cortex distance collapsed across all measurement sites, showing a significant main effect of sex (*F*_(1,102.5)_ = 5.78, *p* = 0.018). Males (blue bar) exhibited significantly greater scalp‐to‐cortex distances (*M* = 20.68 mm, SE = 0.28) compared to females (red bar) (*M* = 19.76 mm, SE = 0.26), with a mean difference of 0.92 mm (95% CI: 0.16–1.67). (B) Average scalp‐to‐cortex distance at four anatomical sites (Afz, Fp1, F3, C3), revealing a significant site‐by‐sex interaction (*F*_(3,314.54)_ = 15.4, *p* < 0.001). Brain images below each bar illustrate the approximate location of each measurement site. Post hoc pairwise comparisons showed significant sex differences at Fp1 (males: 22.28 mm vs. females: 19.77 mm; difference = 2.51 mm, *p* < 0.001) and C3 (males: 19.63 mm vs. females: 18.44 mm; difference = 1.19 mm, *p* = 0.006), but not at Afz (difference = −0.15 mm, *p* = 0.838) or F3 (difference = 0.11 mm, *p* = 0.706). Blue bars represent males; red bars represent females. Error bars represent ±1 SE. *p < 0.05, ***p < 0.001. See Figure S3 for boxplots illustrating the full data distribution.

A significant main effect of sex was observed, *F*_(1,102.5)_ = 5.78, *p* = 0.018 (Figure 2A), with males showing significantly larger scalp‐to‐cortex distances (*M* = 20.68 mm, SE = 0.28) compared to females (*M* = 19.76 mm, SE = 0.26) averaged across all sites. The mean difference was 0.92 mm (95% CI: 0.16–1.67).

A significant site‐by‐sex interaction was found, *F*_(3,314.54)_ = 15.4, *p* < 0.001 (Figure 2B), indicating that sex differences varied across anatomical locations. Post hoc pairwise comparisons revealed the largest sex differences were observed at Fp1, *F*_(2,106)_ = 1.601, *p* < 0.001 (males: 22.28 mm vs. females: 19.77 mm; difference = 2.51 mm), and C3, *F*_(2,106)_ = 0.447, *p* = 0.006 (males: 19.63 mm vs. females: 18.44 mm; difference = 1.19 mm). Sex differences were not significant at Afz, *F*_(2,106)_ = 0.511, *p* = 0.838 (difference = −0.15 mm) or F3, *F*_(2,106)_ = 0.834, *p* = 0.706 (difference = 0.11 mm). For transparency, boxplots illustrating the full data distribution are shown in Figure S3.

**FIGURE 3:**
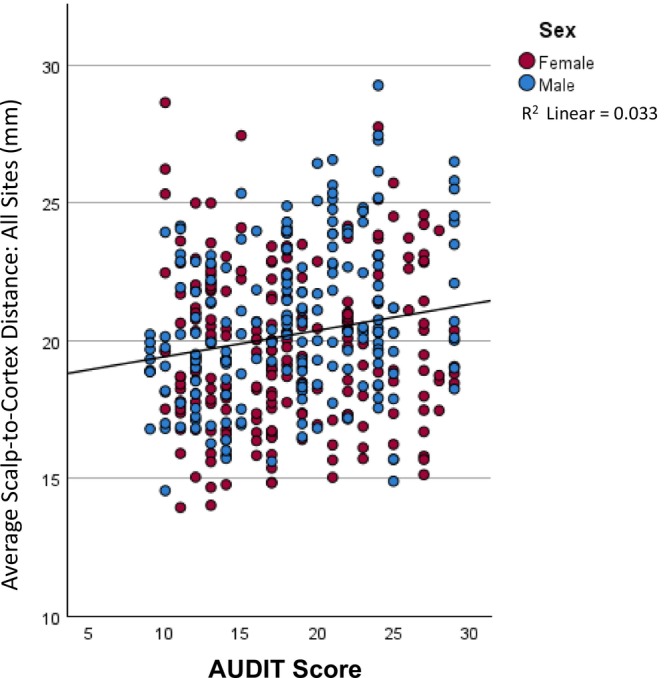
Relationship between alcohol use and scalp‐to‐cortex distance. Scatterplot showing the association between Alcohol Use Disorders Identification Test (AUDIT) scores and average scalp‐to‐cortex distance across all four TMS sites. Each point represents an individual participant, with males shown in blue and females in red. AUDIT score was a significant predictor of scalp‐to‐cortex distance (*F*_(1,104.9)_ = 7.16, *p* = 0.009), with higher AUDIT scores associated with larger scalp‐to‐cortex distances (*R* ^2^ = 0.033). Age was not a significant predictor of scalp‐to‐cortex distance (*F*_(1,107.6)_ = 1.85, *p* = 0.176). The black line represents the linear regression fit across both sexes.

AUDIT score was a significant predictor of overall scalp‐to‐cortex distance, *F*_(1,104.9)_ = 7.16, *p* = 0.009 (Figure 3), with higher AUDIT scores associated with larger distances. Age was not a significant predictor, *F*_(1,107.6)_ = 1.85, *p* = 0.176.

#### 3.2.1 Model Fit

The linear mixed‐effects model demonstrated good fit to the data (marginal *R* ^2^ = 0.305, conditional *R* ^2^ = 0.693). The intraclass correlation coefficient was 0.558, indicating that 55.8% of the variance in scalp‐to‐cortex distance was attributable to between‐participant differences.

### 3.3 Secondary Analysis: Electric Field Modeling

**FIGURE 4:**
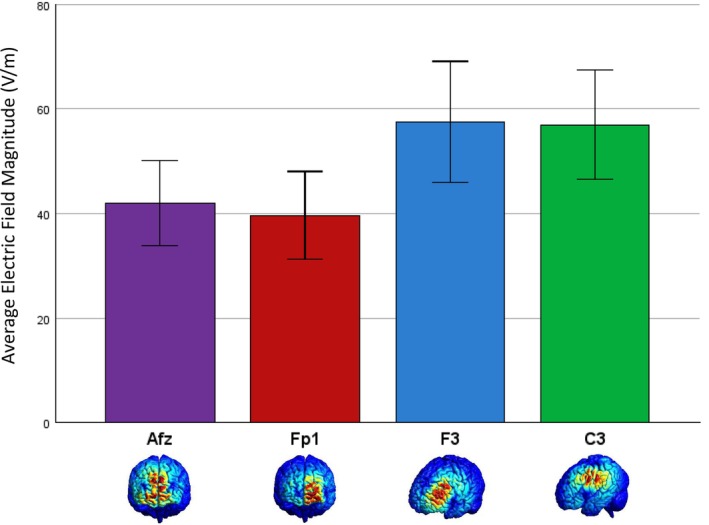
Electric field magnitude varies significantly across TMS sites. Average electric field magnitude (99th percentile value) calculated using SIMNIBS for individual participants at four anatomical sites (Afz, Fp1, F3, C3). Brain images below each bar illustrate the approximate location of each measurement site. There was a significant main effect of TMS site on electric field magnitude (*F*_(3,311.78)_ = 558.66, *p* < 0.001). Post hoc pairwise comparisons revealed significant differences between all sites (*p* < 0.001) except F3 and C3 (*p* > 0.999). Mean electric field magnitude values were: Afz = 41.94 V/m (SE = 0.95), Fp1 = 39.63 V/m (SE = 0.95), F3 = 57.55 V/m (SE = 0.95), and C3 = 56.91 V/m (SE = 0.95). Error bars represent ±1 SE. See Figure S4B for boxplots illustrating the full data distribution.

There was a significant main effect of TMS site on electric field magnitude (99th percentile value), *F*_(3,311.78)_ = 558.66, *p* < 0.001 (Figure 4). Post hoc pairwise comparisons revealed significant differences between all sites (*p* < 0.001) except F3 and C3 (*p* > 0.999). Mean electric field magnitude values were Afz = 41.94 V/m (SE = 0.95), Fp1 = 39.63 V/m (SE = 0.95), C3 = 56.91 V/m (SE = 0.95), and F3 = 57.55 V/m (SE = 0.95). There was no significant effect of sex (*p* = 0.899) nor a sex‐by‐site interaction (*p* = 0.785). For transparency, boxplots illustrating the full data distribution are shown in Figure S2B.

**FIGURE 5:**
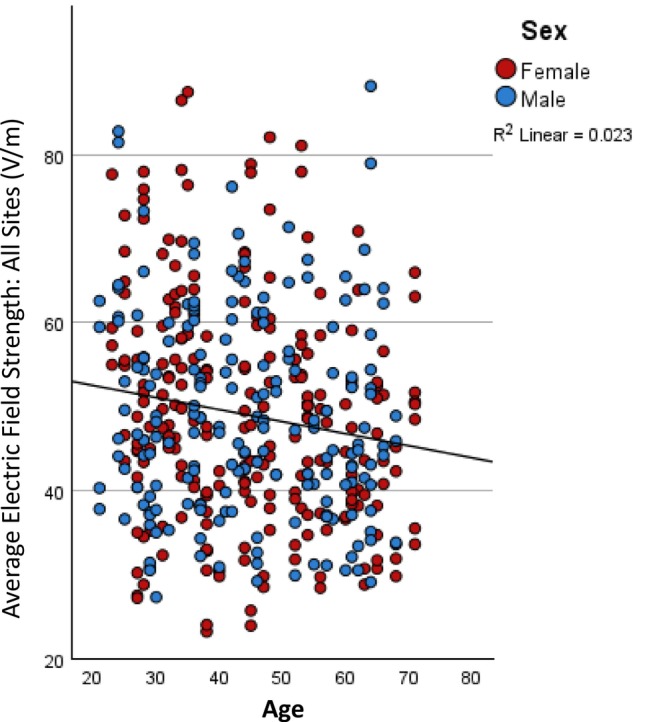
Relationship between age and electric field magnitude. Scatterplot showing the association between age and average electric field magnitude (99th percentile value) across all four TMS sites. Each point represents an individual participant, with males shown in blue and females in red. Age was a significant predictor of electric field magnitude (*F*_(1,117.75)_ = 7.11, *p* = 0.009, *R* ^2^ = 0.023), with older individuals exhibiting lower electric field magnitude. The black line represents the linear regression fit across both sexes.

Age was a significant predictor of electric field magnitude, *F*_(1,117.75)_ = 7.11, *p* = 0.009 (Figure 5), with older individuals exhibiting a decline in electric field magnitude. AUDIT score was not a significant covariate, *F*_(1,109.2)_ = 0.177, *p* = 0.675.

#### 3.3.1 Model Fit

The linear mixed‐effects model demonstrated a good fit to the data (marginal *R* ^2^ = 0.440, conditional *R* ^2^ = 0.898). The intraclass correlation coefficient was 0.818, indicating that 81.8% of the variance in the electric field magnitude 99th percentile value was attributable to between‐participant differences.

### 3.4 Exploratory Analysis

Although the sex‐by‐age interaction for modeled electric field magnitude was not statistically significant in the primary mixed‐effects model (*p* = 0.307), we conducted exploratory, descriptive analyses to visualize potential age‐related patterns stratified by sex. These analyses were not intended to test formal moderation effects but rather to characterize variability that might inform future hypothesis‐driven work. Given the post hoc and exploratory nature of these analyses, all findings should be interpreted cautiously and not as confirmatory evidence of sex‐specific age effects.

In the female‐only model, age was a significant predictor of electric field magnitude (*F*_(1,73.25)_ = 6.79, *p* = 0.011), and site effects remained significant (*F*_(3,173.9)_ = 328.42, *p* < 0.001) (Figure S4). In contrast, the male‐only model showed no significant effect of age (*F*_(1,44.00)_ = 0.825, *p* = 0.369), though site effects were again significant (*F*_(3,138.00)_ = 241.31, *p* < 0.001).

As a further exploratory step, we examined potential moderation of the AUDIT score by adding an age‐by‐AUDIT interaction term. In females, this interaction approached significance (*F*_(1,57.90)_ = 3.758, *p* = 0.057) (Figure S5). However, no statistically significant interaction was observed, and these subgroup patterns were not subjected to confirmatory hypothesis testing. Accordingly, these observations should be viewed as descriptive and hypothesis‐generating only. No significant effects were observed in the male‐only models.

## 4 Discussion

The present study examined anatomical variability in STC distance and modeled electric field magnitude across four common TMS target sites in individuals with AUD, with a focus on sex differences, age, and alcohol use severity.

### 4.1 Key Findings

Our findings revealed significant main effects of TMS target site, sex, and AUDIT score on scalp‐to‐cortex distance, as well as a significant site‐by‐sex interaction. Specifically, males exhibited significantly greater STC distances than females at Fp1 (left vmPFC), consistent with prior findings in AUD, and C3 (motor cortex), extending sex‐related STC differences to an additional region in an AUD population. In contrast, no sex differences were observed at Afz (mPFC) or F3 (DLPFC), consistent with region‐specific patterns reported in prior STC studies of healthy and aging populations.

Despite these anatomical differences, modeled electric field magnitude did not differ significantly by sex. However, a robust site effect emerged, with significantly higher electric field magnitudes observed at C3 and F3 compared to Afz and Fp1. This suggests that while motor cortex‐based dosing may be appropriate for lateral targets like F3, it may be inadequate for midline regions such as Afz and Fp1, which consistently exhibited lower electric field values. These findings underscore the need for individualized dosing strategies that account for anatomical variability across cortical targets in individuals with AUD, especially when targeting non‐motor regions.

Age emerged as a significant predictor of electric field magnitude, with older individuals exhibiting lower estimated electric field magnitude at the cortex, consistent with studies demonstrating age‐related anatomical variability with important implications for effective field strength (Van Hoornweder et al. 2024). Interestingly, while age was associated with electric field magnitude, it did not significantly predict STC distance. This suggests that age‐related reductions in modeled electric field magnitude may reflect neuroanatomical changes beyond cortical depth alone.

From a current‐flow modeling perspective, finite‐element models account for the conductive properties and spatial distribution of head tissues, and age‐related anatomical changes can therefore substantially influence electric field propagation. For example, increases in cerebrospinal fluid volume with age may result in greater current shunting away from cortical gray matter due to the high conductivity of cerebrospinal fluid, thereby reducing the electric field magnitude reaching the cortex. Similarly, age‐related alterations in tissue composition and conductivity, as well as changes in white matter integrity, may modify current pathways and local field distributions within the brain. Together, these factors provide a plausible biophysical explanation for age‐related reductions in modeled electric field magnitude that extend beyond simple measures of STC distance.

Though the sex‐by‐age interaction in the primary model was not statistically significant, visual inspection and exploratory sex‐stratified analyses suggested the age‐related decline in modeled electric field magnitude (i.e., reduced stimulation intensity reaching the cortex with increasing age) may be more pronounced in females, particularly at MPFC sites. In post hoc female‐only models, an interaction between age and AUDIT score approached significance, raising the possibility that alcohol use severity could moderate age‐related declines in this group. In contrast, no significant effects of age or AUDIT score were observed in males. However, given the exploratory nature of these stratified analyses and the non‐significant interaction in the primary model, these patterns require replication in adequately powered studies before firm conclusions can be drawn regarding sex‐specific vulnerabilities to TMS efficacy.

Taken together, these results highlight the need for personalized TMS dosing strategies that account for anatomical variability, sex, and age. Our findings extend prior STC and electric‐field modeling work conducted in healthy and aging samples (Lu et al. 2021; McCalley et al. 2026; Van Hoornweder et al. 2024) by demonstrating how sex, age, and alcohol use severity relate to anatomical variability in an AUD population. In this context, traditional motor cortex‐based dosing may not generalize well to non‐motor targets in individuals with AUD, and sex‐specific anatomical and neurobiological factors may influence stimulation outcomes in underrepresented populations such as older females with AUD.

Although the present findings may have implications for treatment optimization, they should not be interpreted to mean that stimulation intensity should simply be increased above standard motor‐threshold‐based dosing on the basis of sex alone. Rather, greater STC distance may contribute to inter‐individual differences in the effective cortical dose delivered by conventional protocols, particularly when motor‐cortex‐based dosing is applied to non‐motor targets. These data therefore support the potential value of individualized, target‐specific dosing approaches, including more precise threshold estimation, MRI‐informed targeting, and electric‐field modeling. Importantly, any attempt to optimize dosing must remain within established TMS safety guidelines, especially in individuals with factors that lower seizure threshold, such as acute alcohol withdrawal. While sex has been proposed as a moderator of neuromodulation response more broadly, the existing TMS literature in AUD has not yet demonstrated clear and consistent sex differences in treatment outcome. Accordingly, the present results should be viewed as mechanistic evidence that may help guide future sex‐stratified clinical trials rather than as evidence supporting sex‐based dose escalation in current practice.

### 4.2 Sex‐Based Differences in STC Distance

Consistent with prior work (Hanlon and McCalley 2022; McCalley and Hanlon 2021), males with AUD showed significantly greater STC distances than females at Fp1. While earlier studies did not report significant sex differences at the motor cortex, this may have been due to limited female representation. In contrast, our more balanced sample revealed that females with AUD have significantly smaller STC distances at C3. Interestingly, no sex differences in STC distance were observed at Afz or F3, the two sites currently being targeted in our ongoing clinical trial. While F3 represents a lateral prefrontal target, Afz corresponds to an MPFC site, a region increasingly implicated in addiction‐related processes. Although Afz exhibited the largest overall STC distance among the targets examined, the absence of sex‐dependent anatomical variability at this site suggests that MPFC stimulation via Afz may be relatively consistent across sexes from a structural and biophysical perspective. Importantly, this observation does not imply superior or established treatment efficacy, nor does it mitigate the potential impact of greater absolute cortical depth on effective stimulation strength. Rather, these findings highlight Afz as a midline MPFC target with reduced sex‐related anatomical variability, which may be advantageous for mechanistic studies and for evaluating sex‐neutral dosing considerations should efficacy be demonstrated in future outcome‐based neuromodulation trials.

Although males had greater STC distances at the motor cortex, no significant sex differences were found in rMT, suggesting that these anatomical differences may not directly translate to differences in motor cortex‐based dosing. This discrepancy highlights a limitation of relying solely on rMT for determining stimulation intensity at other cortical sites. Relying solely on motor cortex‐based rMT for dosing may lead to suboptimal stimulation, especially in regions like Afz and Fp which showed lower modeled electric field magnitudes.

### 4.3 Alcohol Use Severity and STC Distance

Higher AUDIT scores were associated with greater STC distances, supporting prior literature suggesting that alcohol‐related neurodegeneration contributes to anatomical changes that may impact TMS efficacy. Chronic alcohol use has been linked to cortical thinning and gray matter volume loss, particularly in prefrontal regions (Cardenas et al. 2007; Durazzo et al. 2008; Zahr et al. 2017). These structural changes may increase the physical distance between the scalp and cortex, thereby attenuating the strength of TMS‐induced electric fields (McCalley and Hanlon 2021). Importantly, this relationship remained significant even after controlling for age, suggesting that alcohol use severity may be a more salient predictor of STC variability than chronological aging alone.

### 4.4 Age‐Related Decline and Sex‐Specific Vulnerability

Age emerged as a significant predictor of electric field magnitude, with older individuals with AUD exhibiting reduced electric fields, consistent with age‐related anatomical effects described in prior modeling studies. In exploratory, sex‐stratified analyses, age‐related reductions in electric field magnitude appeared visually steeper in females, particularly at medial prefrontal targets; however, the sex‐by‐age interaction was not statistically significant in the primary model, and these observations should be interpreted cautiously. Although speculative, several existing frameworks may help contextualize these exploratory patterns. The telescoping hypothesis proposes that females progress more rapidly through the stages of AUD and suffer more severe neurobiological consequences over the lifespan (Kirsch et al. 2025; Piazza et al. 1989). Consistent with this framework, neuroimaging studies have confirmed that females are more susceptible to alcohol‐related cortical atrophy, even when controlling for consumption levels (Cornish and Prasad 2021; Verplaetse et al. 2021). Despite comparable AUDIT scores between sexes in the present study, older females may exhibit greater structural brain deterioration, which could contribute to diminished TMS efficacy due to reduced cortical excitability and increased STC distance (Hanlon and McCalley 2022). Moreover, biological factors such as lower levels of estradiol in aging women may further reduce cortical responsiveness to stimulation, compounding the effects of anatomical changes (Hanlon and McCalley 2022). Taken together, these considerations suggest that age and sex may jointly shape anatomical and neurobiological factors relevant for TMS engagement, but the present findings are best viewed as hypothesis‐generating and warrant replication in larger, adequately powered samples.

### 4.5 Exploratory Models and Moderation by AUD Severity

Although the sex‐by‐age interaction was not statistically significant in the primary model, we conducted sex‐stratified analyses given our a priori interest in potential sex differences. In the female‐only model, age significantly predicted electric field magnitude, whereas no significant age effect was observed in the male‐only model. Additionally, in females, the AUDIT score was a significant predictor of electric field magnitude, and the age‐by‐AUDIT interaction approached significance. No significant effects of age or AUDIT score were observed in males.

These findings suggest potential sex‐specific patterns in how age and alcohol use severity relate to electric field magnitude. Specifically, exploratory analyses suggested that alcohol use severity may be associated with qualitatively different age‐related patterns of modeled electric field magnitude among females with AUD, a pattern that was not observed in males. This suggests potential sex‐specific vulnerability to reduced TMS‐induced electric fields and highlights the possible need for sex‐tailored dosing strategies. However, given the non‐significant sex‐by‐age interaction in the primary model, these stratified results should be interpreted cautiously and require replication in future studies.

### 4.6 Limitations and Future Directions

This study has several limitations that should be considered when interpreting the findings. First, the absence of a healthy control group limits the ability to determine whether the observed variability in STC distance and its association with alcohol use severity reflect AUD‐specific neuroanatomical changes or general population variability. Although our sample was balanced by sex, it was predominantly composed of white participants, which may limit the generalizability of our results to more racially and ethnically diverse populations. While sex is a biological variable, race is a social construct without a biological basis; however, it remains important to study due to the impact of systemic inequities on health outcomes, which may contribute to variability in treatment efficacy. Future research should prioritize recruiting more representative samples and examining how race, ethnicity, and other demographic factors influence STC variability and electric field modeling.

In addition, the present analyses were limited by the absence of detailed lifetime drinking history measures, such as age of AUD onset or cumulative duration of heavy drinking. While AUDIT provides a well‐validated index of alcohol use severity and related consequences, it primarily reflects current symptom severity rather than cumulative ethanol exposure across the lifespan. As a result, we were unable to examine whether indices of drinking chronicity or duration differentially relate to STC distance or modeled electric field magnitude. Future studies incorporating age‐of‐onset measures or longitudinal drinking histories will be important for disentangling the relative contributions of recent alcohol use, long‐term exposure, and chronological aging to neuroanatomical variability relevant for TMS dosing.

In the present study, rMT was determined using visual observation rather than electromyography (EMG), which remains the gold standard for TMS threshold estimation (Barker et al. 1985). Visual methods introduce observer‐related variability and can yield thresholds that differ from EMG‐based estimates by approximately −10% to +2% of maximal stimulator output (Balslev et al. 2007; Hamoline et al. 2025; Pridmore et al. 1998). This discrepancy may lead to inaccurate dosing, particularly when targeting non‐motor regions where precise stimulation is critical. While EMG provides more sensitive and reliable measurements, it is not always feasible in clinical or large‐scale research settings. In such cases, accelerometry has emerged as a promising alternative (Hamoline et al. 2024), offering greater objectivity than visual observation. Future studies should consider integrating EMG or accelerometry based methods to enhance dosing precision, especially in populations with AUD where anatomical variability may further complicate stimulation accuracy.

In addition, finite‐element head models were constructed using T1‐weighted structural MRI data alone. Although the inclusion of both T1‐ and T2‐weighted images is considered best practice for optimizing tissue segmentation and improving the accuracy of boundary definitions, particularly for cerebrospinal fluid and skull compartments (Nielsen et al. 2018), T2‐weighted images were not available for all participants in this dataset. Because the primary anatomical measure examined in the present study was STC distance, rather than fine‐grained tissue conductivity boundaries, the impact of this limitation is likely modest for the current analyses. Nevertheless, future studies incorporating both T1‐ and T2‐weighted imaging will be important for improving model precision and for extending these findings to more detailed investigations of tissue‐specific contributions to electric field propagation.

While STC distance is a critical anatomical factor in electric field modeling, it is not the only one. Differences in gray matter volume and gyral folding complexity, both of which show sex‐specific patterns in healthy populations, may also influence electric field distribution. For example, females tend to have greater cortical thickness and gyral surface area in certain regions, while males show larger volumes in subcortical and motor‐related areas (Diaz‐Caneja et al. 2021; Lotze et al. 2019; Sanchis‐Segura et al. 2019). These structural features may interact with STC distance to shape stimulation outcomes, and future studies should incorporate high‐resolution morphometric analyses to better understand these relationships.

Additionally, longitudinal studies are needed to determine whether reductions in alcohol consumption lead to measurable changes in STC distance and cortical structure, and whether such recovery differs by sex. Preliminary evidence suggests that abstinence may lead to partial recovery of brain network connectivity (van Oort et al. 2023), but it remains unclear whether these functional changes are accompanied by structural improvements in STC distance or cortical volume. Investigating how STC distance varies across stages of disease progression (e.g., mild, moderate, severe AUD) could provide critical insights into identifying optimal candidates and timing for TMS interventions. Future research should explore whether the magnitude and rate of structural recovery differ by sex during periods of reduced drinking or abstinence. Given the observed sex‐specific vulnerabilities to alcohol‐related neurodegeneration, clarifying whether males and females show different patterns of structural brain recovery could inform adaptive dosing strategies and improve treatment efficacy for both males and females with AUD. Finally, while the present analyses were intended to characterize associations between demographic factors, anatomical variability, and modeled electric field magnitude, future studies with larger or longitudinal samples could employ formal mediation or structural equation modeling approaches to test whether STC distance or other anatomical features statistically mediate the effects of age, sex, or alcohol use severity on effective stimulation dose.

## 5 Conclusion

Overall, these findings underscore the importance of personalized TMS protocols that account for anatomical variability, sex, and age in individuals with AUD. The observed differences in STC distance and electric field magnitude across cortical sites challenge the adequacy of traditional motor cortex‐based dosing approaches, particularly for non‐motor targets like Afz and Fp1. Given the underrepresentation of females in prior TMS studies, our results emphasize the need for more balanced recruitment and sex‐stratified analyses in future trials. Moreover, the sex‐specific effects of age and alcohol use severity on modeled electric field magnitude highlight the potential for tailored interventions, especially for older females who may be more vulnerable to alcohol‐related neurodegeneration. As TMS continues to evolve as a therapeutic modality for AUD, integrating individualized anatomical and demographic factors will be critical for optimizing clinical outcomes.

## Author Contributions

Kaitlin R. Kinney conceptualized the present data analysis study, conducted all data and statistical analyses, and prepared the initial manuscript draft with important intellectual input from Merideth A. Addicott. Kaitlin R. Kinney, Nathanial E. Stewart, Michiyah Kimber, Hannah E. DeMaioNewton and Jazmyne S. James contributed to participant recruitment, data collection, resource management, and provided critical review of the manuscript. Nathan R. Luzum contributed to data analysis and provided a critical review of the manuscript. Edward H. Ip provided methodological guidance, statistical consultation, and a critical review of the manuscript. Hilary R. Smith contributed to the study resources and provided a critical review of the manuscript. Drew D. Kiraly served as the study physician and provided a critical review of the manuscript. Colleen A. Hanlon conceptualized the original study protocol and acquired funding for the parent clinical trial. Merideth A. Addicott contributed to conceptualization, project administration, and provided critical review of the manuscript. All authors reviewed and approved the final version of the manuscript.

## Funding

This work was supported by the National Institutes of Health, National Institute on Alcohol Abuse and Alcoholism, R01 AA027705, T32 AA007565, P50 AA026117.

## Conflicts of Interest

Dr. Colleen Hanlon has a financial interest in BrainsWay Ltd. The other authors declare no conflicts of interest.

## Supporting information


**Data S1:** acer70358‐sup‐0001‐DataS1.docx.

**Figure S1:** Representative electric field distribution patterns across TMS sites and sexes. Electric field magnitude distributions for representative male (♂) and female (♀) participants at four cortical target sites: Afz (medial prefrontal cortex, MPFC), Fp1 (ventromedial prefrontal cortex, vmPFC), F3 (dorsolateral prefrontal cortex, DLPFC), and C3 (motor cortex). Each model represents a unique individual selected to illustrate the range of electric field magnitudes observed across participants. For each site and sex, three representative cases are shown displaying strong (top row), medium (middle row), and weak (bottom row) electric field patterns. Electric field maps were generated using SIMNIBS and are shown in subject‐space. The color scale represents electric field magnitude in volts per meter (V/m), ranging from 0 (blue) to 100 (red).

**Figure S2:** Distribution of scalp‑to‑cortex distance and modeled electric field magnitude across stimulation sites.

**Figure S3:** Sex‑specific distributions of scalp‑to‑cortex distance across stimulation sites.

**Figure S4:** Sex‐specific relationships between age and electric field magnitude. Scatterplot showing the association between age and average electric field magnitude (99th percentile value) across all four TMS sites, with separate regression lines for males (blue) and females (red). Each point represents an individual participant. Visual inspection suggested differing age‑related patterns across sexes, with age‑related reductions in electric field magnitude more apparent within females, particularly at older ages. Post‐hoc sex‐stratified linear mixed‐effects models revealed that age was a significant predictor of electric field magnitude in females (*F*_(1,73.25)_ = 6.79, *p* = 0.011, *R* ^2^ = 0.036) but not in males (*F*_(1,44.00)_ = 0.825, *p* = 0.369, *R* ^2^ = 0.010). Note that the sex by age interaction was not statistically significant in the full model (*p* = 0.307). Accordingly, these sex‑stratified patterns are exploratory and should be interpreted cautiously.

**Figure S5:** Exploratory analysis of age, alcohol use, and electric field magnitude by sex. Average electric field magnitude (99th percentile value) across all four TMS sites, stratified by sex, age group, and AUDIT score category. Left panel shows females (♀); right panel shows males (♂). Participants are grouped into moderate alcohol dependence (AUDIT scores 9–19, blue bars) and severe alcohol dependence (AUDIT scores 20+, red bars) categories across five age bins (≤ 30, 31–40, 41–50, 51–60, 61–71 years). Error bars represent ±1 SE. In females, exploratory analyses indicated qualitatively different age‑related profiles of electric field magnitude across AUDIT categories. A post‑hoc age by AUDIT interaction term did not reach statistical significance (*F*_(1,57.90)_ = 3.758, *p* = 0.057), and AUDIT score was a significant predictor in the female‑only model (*F*_(1,60.64)_ = 4.78, *p* = 0.033). No statistically significant effects of age, AUDIT score, or their interaction were observed in the male‑only models. All sex‑stratified and AUDIT‑stratified findings shown here are exploratory and should be interpreted cautiously.

## Data Availability

The data that support the findings of this study are available from the corresponding author upon reasonable request.
