# Supplementary material for: Sex Differences in Scalp‐to‐Cortex Distance: Implications for Transcranial Magnetic Stimulation Efficacy in Alcohol Use Disorder
Source: Alcohol Clin Exp Res (Hoboken). 2026 Jun 30;50(7):e70358. doi: 10.1111/acer.70358 (PMC13316460; doi:10.1111/acer.70358)
Supplement: Supplementary file 1 — Data S1: acer70358‐sup‐0001‐DataS1.docx. [file ACER-50-0-s003.docx]

**Supplementary Figure 1. Representative electric field distribution patterns across TMS sites and sexes.** Electric field magnitude distributions for representative male (♂) and female (♀) participants at four cortical target sites: Afz (medial prefrontal cortex, MPFC), Fp1 (ventromedial prefrontal cortex, vmPFC), F3 (dorsolateral prefrontal cortex, DLPFC), and C3 (motor cortex). Each model represents a unique individual selected to illustrate the range of electric field magnitudes observed across participants. For each site and sex, three representative cases are shown displaying strong (top row), medium (middle row), and weak (bottom row) electric field patterns. Electric field maps were generated using SIMNIBS and are shown in subject-space. The color scale represents electric field magnitude in volts per meter (V/m), ranging from 0 (blue) to 100 (red).

**Supplementary Figure 2. Distribution of scalp‑to‑cortex distance and modeled electric field magnitude across stimulation sites.**

(A) Boxplots showing the distribution of scalp‑to‑cortex (STC) distance (mm) for each stimulation site (Afz, Fp1, F3, C3). (B) Boxplots showing the distribution of modeled electric field magnitude (V/m; 99th percentile value) for each stimulation site.

For all boxplots, the central line represents the median, boxes indicate the interquartile range, and whiskers denote the data range excluding outliers. These plots are provided to visualize inter‑individual variability underlying the summary statistics reported in the main figures.

**Supplementary Figure 3. Sex‑specific distributions of scalp‑to‑cortex distance across stimulation sites.**

Boxplots show the distribution of scalp‑to‑cortex (STC) distance (mm) for each stimulation site (Afz, Fp1, F3, C3), displayed separately for males (♂) and females (♀). The central line represents the median, boxes indicate the interquartile range, and whiskers denote the data range excluding outliers. Asterisks indicate statistically significant sex differences at the corresponding stimulation sites, consistent with the site by sex interaction reported in the main analyses.

**Supplementary Figure 4. Sex-specific relationships between age and electric field magnitude.** Scatterplot showing the association between age and average electric field magnitude (99th percentile value) across all four TMS sites, with separate regression lines for males (blue) and females (red). Each point represents an individual participant. Visual inspection suggested differing age‑related patterns across sexes, with age‑related reductions in electric field magnitude more apparent within females, particularly at older ages. Post-hoc sex-stratified linear mixed-effects models revealed that age was a significant predictor of electric field magnitude in females (*F*_(1, 73.25)_ = 6.79, *p* = 0.011, R² = 0.036) but not in males (*F*_(1, 44.00)_ = 0.825, *p* = 0.369, R² = 0.010). Note that the sex by age interaction was not statistically significant in the full model (*p* = 0.307). Accordingly, these sex‑stratified patterns are exploratory and should be interpreted cautiously.

**Supplementary Figure 5. Exploratory analysis of age, alcohol use, and electric field magnitude by sex.** Average electric field magnitude (99th percentile value) across all four TMS sites, stratified by sex, age group, and AUDIT score category. Left panel shows females (♀); right panel shows males (♂). Participants are grouped into moderate alcohol dependence (AUDIT scores 9-19, blue bars) and severe alcohol dependence (AUDIT scores 20+, red bars) categories across five age bins (≤30, 31-40, 41-50, 51-60, 61-71 years). Error bars represent ±1 SE. In females, exploratory analyses indicated qualitatively different age‑related profiles of electric field magnitude across AUDIT categories. A post‑hoc age by AUDIT interaction term did not reach statistical significance (*F*_(1, 57.90)_ = 3.758, *p* = 0.057), and AUDIT score was a significant predictor in the female‑only model (*F*_(1, 60.64)_ = 4.78, *p* = 0.033). No statistically significant effects of age, AUDIT score, or their interaction were observed in the male‑only models. All sex‑stratified and AUDIT‑stratified findings shown here are exploratory and should be interpreted cautiously.
