# Supplementary figures and images for: Sex Differences in Scalp‐to‐Cortex Distance: Implications for Transcranial Magnetic Stimulation Efficacy in Alcohol Use Disorder
Source: Alcohol Clin Exp Res (Hoboken). 2026 Jun 30;50(7):e70358. doi: 10.1111/acer.70358 (PMC13316460; doi:10.1111/acer.70358)

## Slide 1
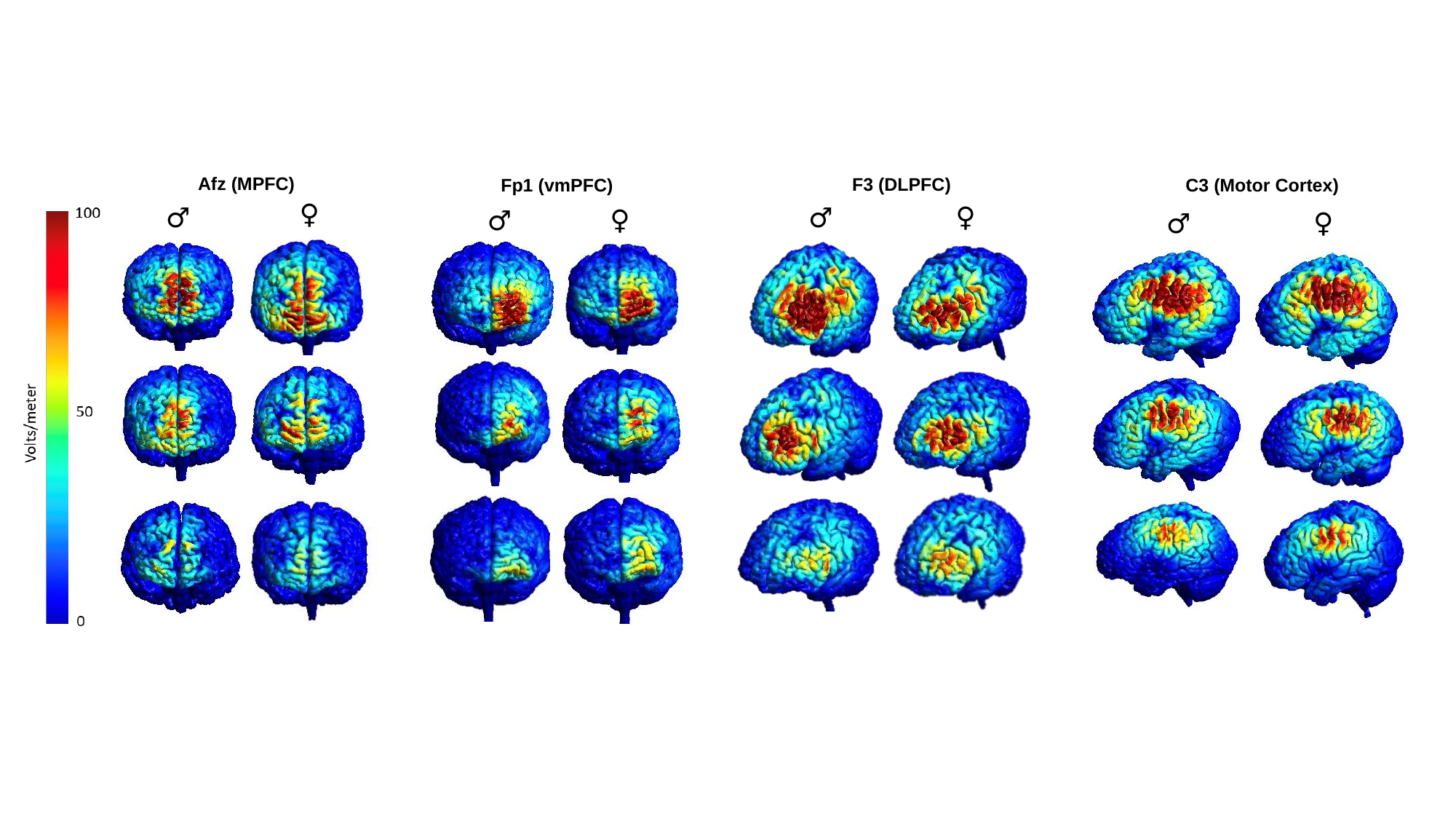

♀
♂
♂
♀
Afz (MPFC)
♀
♂
F3 (DLPFC)
Fp1 (vmPFC)
C3 (Motor Cortex)
♀
♂
♂

Supplement: Supplementary file 2 — Figure S1: Representative electric field distribution patterns across TMS sites and sexes. Electric field magnitude distributions for representative male (♂) and female (♀) participants at four cortical target sites: Afz (medial prefrontal cortex, MPFC), Fp1 (ventromedial prefrontal cortex, vmPFC), F3 (dorsolateral prefrontal cortex, DLPFC), and C3 (motor cortex). Each model represents a unique individual selected to illustrate the range of electric field magnitudes observed across participants. For each site and sex, three representative cases are shown displaying strong (top row), medium (middle row), and weak (bottom row) electric field patterns. Electric field maps were generated using SIMNIBS and are shown in subject‐space. The color scale represents electric field magnitude in volts per meter (V/m), ranging from 0 (blue) to 100 (red). [file ACER-50-0-s006.pptx]

## Slide 1
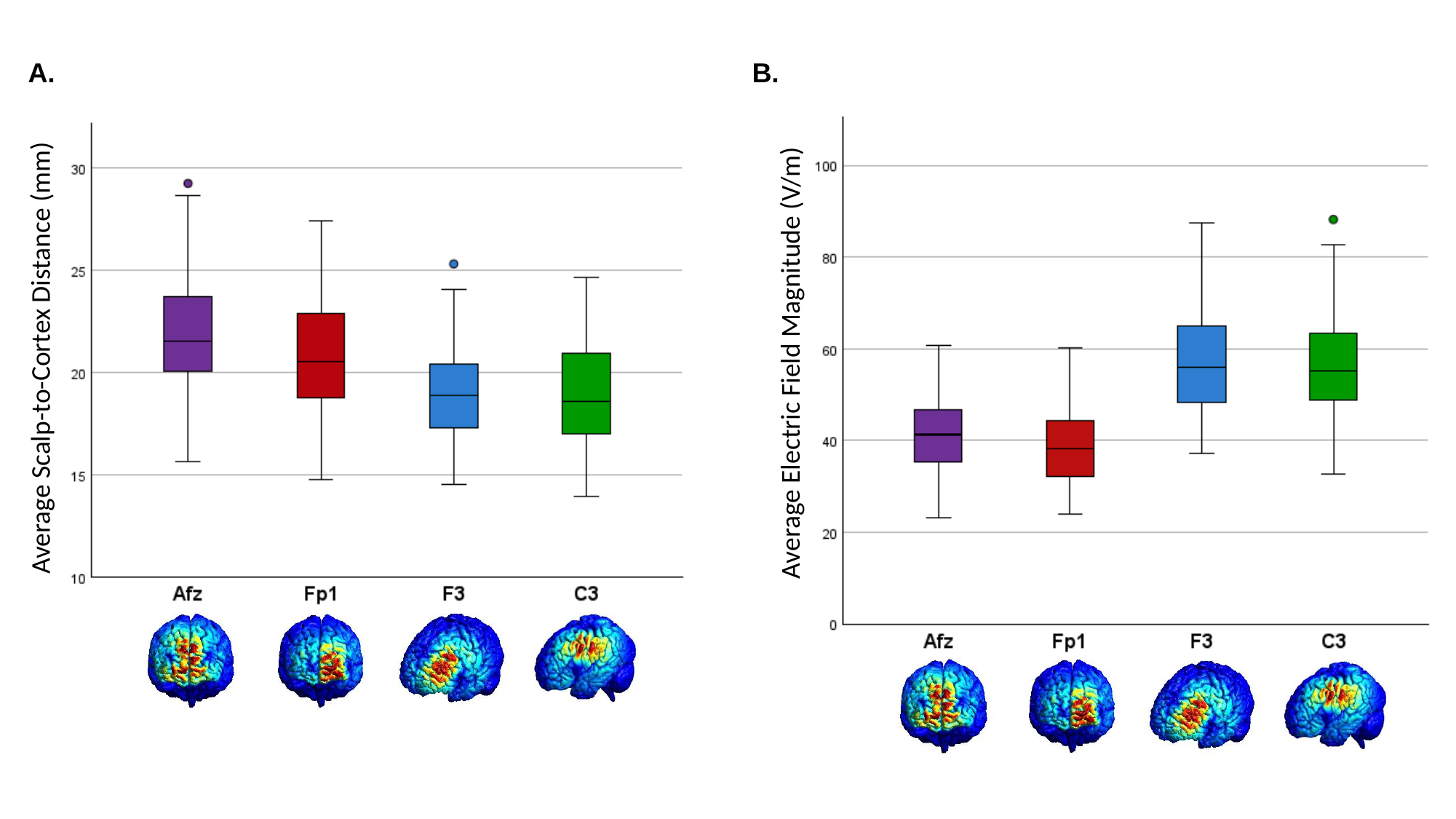

A.
B.
Average Scalp-to-Cortex Distance (mm)
Average Electric Field Magnitude (V/m)

Supplement: Supplementary file 3 — Figure S2: Distribution of scalp‑to‑cortex distance and modeled electric field magnitude across stimulation sites. [file ACER-50-0-s002.pptx]

## Slide 1
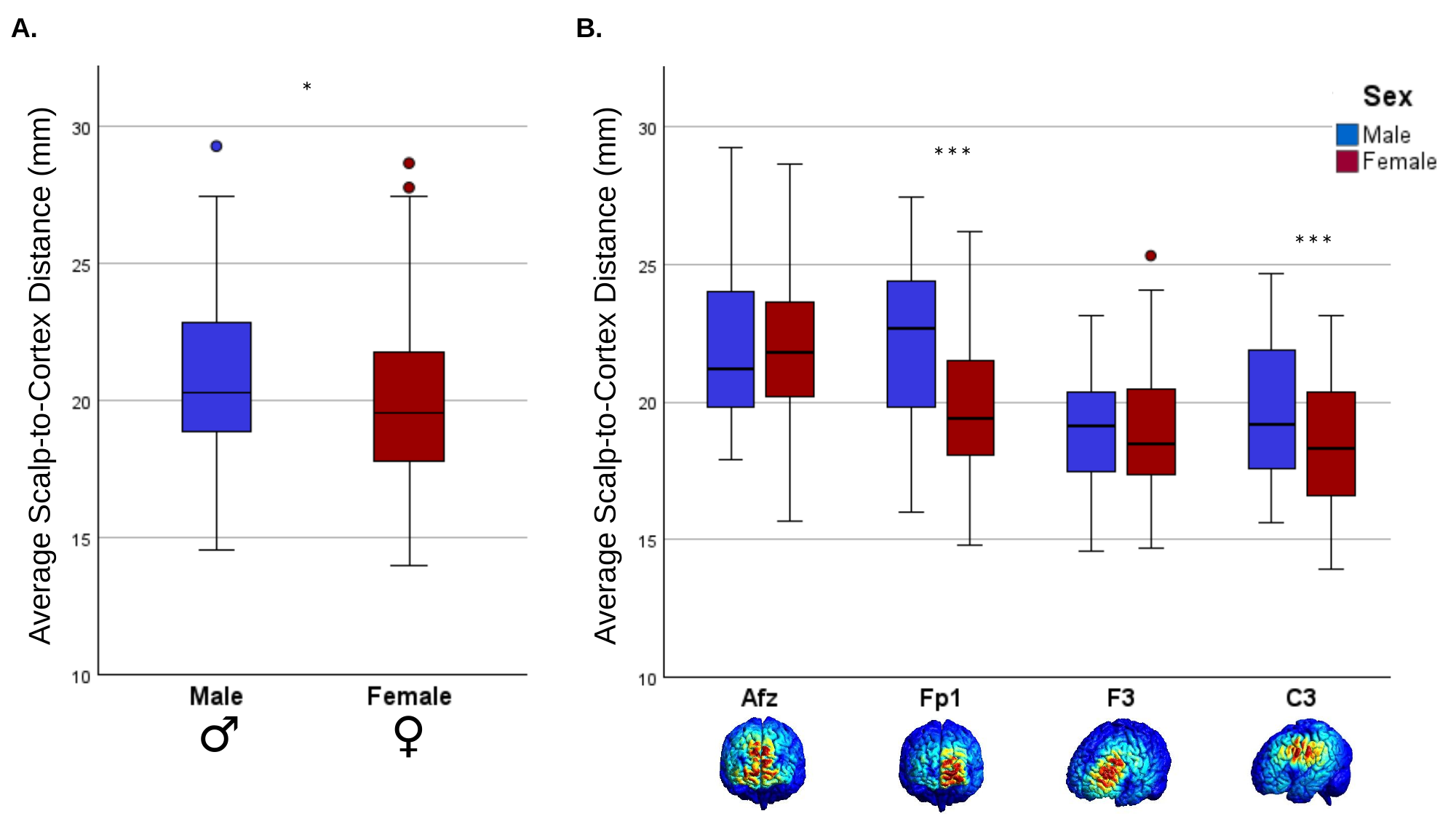

A.
B.
*
***
***
Average Scalp-to-Cortex Distance (mm)
Average Scalp-to-Cortex Distance (mm)
♂
♀

Supplement: Supplementary file 4 — Figure S3: Sex‑specific distributions of scalp‑to‑cortex distance across stimulation sites. [file ACER-50-0-s004.pptx]

## Slide 1
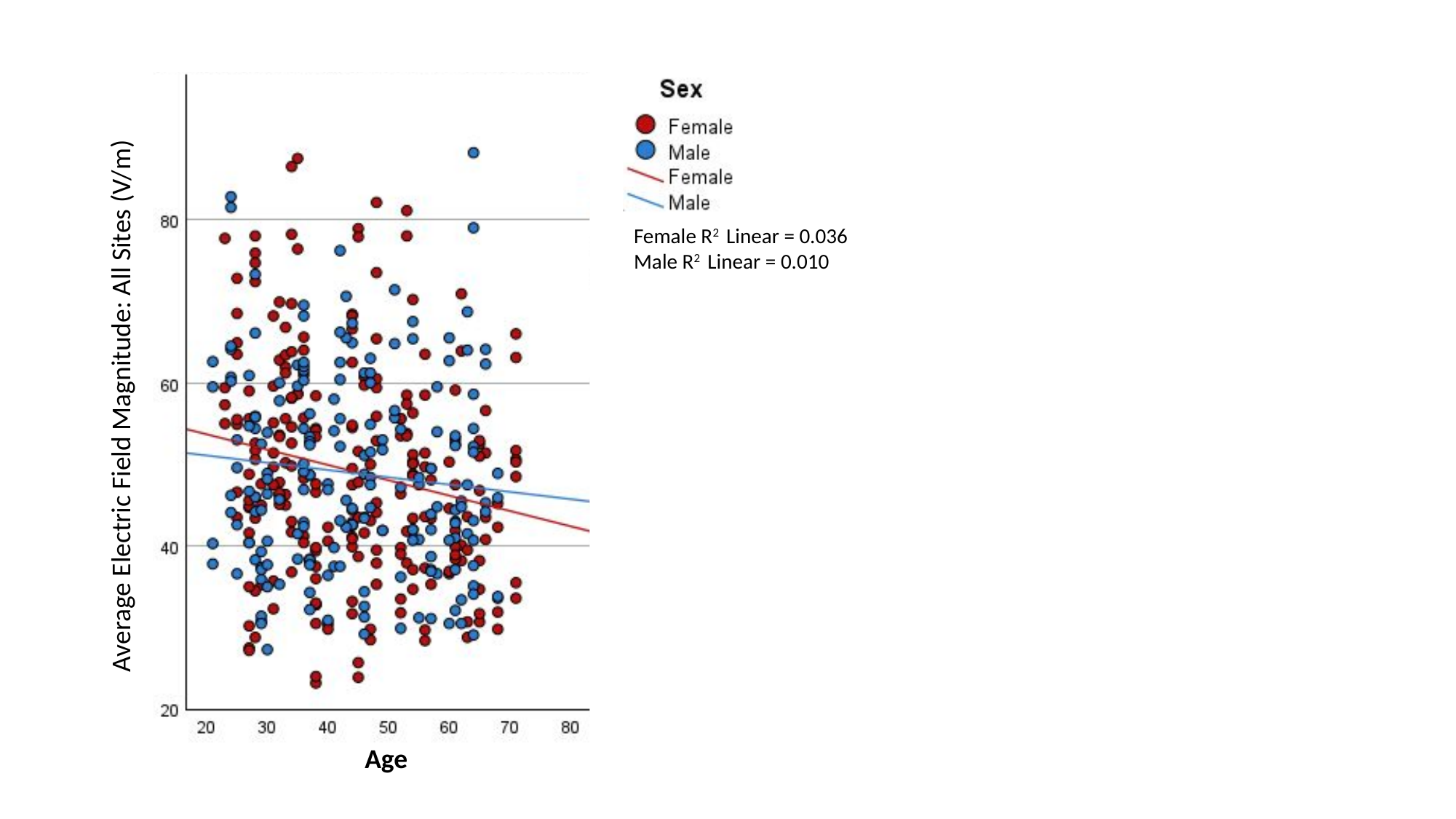

Female R2 Linear = 0.036
Male R2 Linear = 0.010
Average Electric Field Magnitude: All Sites (V/m)
Age

Supplement: Supplementary file 5 — Figure S4: Sex‐specific relationships between age and electric field magnitude. Scatterplot showing the association between age and average electric field magnitude (99th percentile value) across all four TMS sites, with separate regression lines for males (blue) and females (red). Each point represents an individual participant. Visual inspection suggested differing age‑related patterns across sexes, with age‑related reductions in electric field magnitude more apparent within females, particularly at older ages. Post‐hoc sex‐stratified linear mixed‐effects models revealed that age was a significant predictor of electric field magnitude in females (F (1,73.25) = 6.79, p = 0.011, R 2 = 0.036) but not in males (F (1,44.00) = 0.825, p = 0.369, R 2 = 0.010). Note that the sex by age interaction was not statistically significant in the full model (p = 0.307). Accordingly, these sex‑stratified patterns are exploratory and should be interpreted cautiously. [file ACER-50-0-s005.pptx]
