# Supplementary material for: Sex Differences in Scalp‐to‐Cortex Distance: Implications for Transcranial Magnetic Stimulation Efficacy in Alcohol Use Disorder
Source: Alcohol Clin Exp Res (Hoboken). 2026 Jun 30;50(7):e70358. doi: 10.1111/acer.70358 (PMC13316460; doi:10.1111/acer.70358)
Supplement: Supplementary file 6 — Figure S5: Exploratory analysis of age, alcohol use, and electric field magnitude by sex. Average electric field magnitude (99th percentile value) across all four TMS sites, stratified by sex, age group, and AUDIT score category. Left panel shows females (♀); right panel shows males (♂). Participants are grouped into moderate alcohol dependence (AUDIT scores 9–19, blue bars) and severe alcohol dependence (AUDIT scores 20+, red bars) categories across five age bins (≤ 30, 31–40, 41–50, 51–60, 61–71 years). Error bars represent ±1 SE. In females, exploratory analyses indicated qualitatively different age‑related profiles of electric field magnitude across AUDIT categories. A post‑hoc age by AUDIT interaction term did not reach statistical significance (F (1,57.90) = 3.758, p = 0.057), and AUDIT score was a significant predictor in the female‑only model (F (1,60.64) = 4.78, p = 0.033). No statistically significant effects of age, AUDIT score, or their interaction were observed in the male‑only models. All sex‑stratified and AUDIT‑stratified findings shown here are exploratory and should be interpreted cautiously. [file ACER-50-0-s001.pptx]

## Slide 1
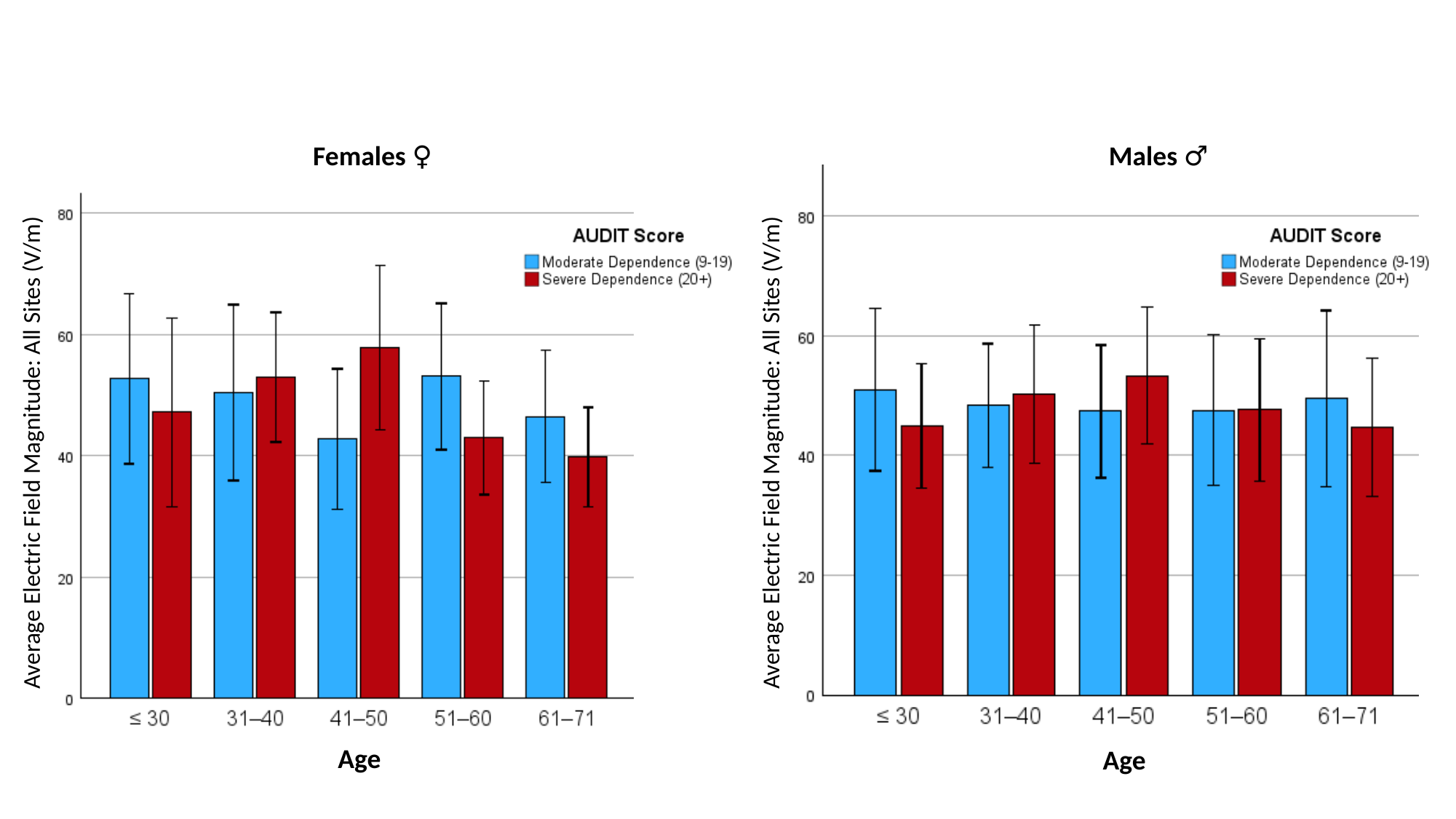

Females ♀
Males ♂
Average Electric Field Magnitude: All Sites (V/m)
Average Electric Field Magnitude: All Sites (V/m)
Age
Age
